# Cochlear implant – state of the art

**DOI:** 10.3205/cto000143

**Published:** 2018-02-19

**Authors:** Thomas Lenarz

**Affiliations:** 1 Department of Otolaryngology, Head & Neck Surgery, Hannover Medical School, Hannover, Germany

**Keywords:** cochlear implant, sensory deafness, diagnostics, surgical procedure, results, complications, future developments

## Abstract

Cochlear implants are the treatment of choice for auditory rehabilitation of patients with sensory deafness. They restore the missing function of inner hair cells by transforming the acoustic signal into electrical stimuli for activation of auditory nerve fibers. Due to the very fast technology development, cochlear implants provide open-set speech understanding in the majority of patients including the use of the telephone. Children can achieve a near to normal speech and language development provided their deafness is detected early after onset and implantation is performed quickly thereafter. The diagnostic procedure as well as the surgical technique have been standardized and can be adapted to the individual anatomical and physiological needs both in children and adults. Special cases such as cochlear obliteration might require special measures and re-implantation, which can be done in most cases in a straight forward way. Technology upgrades count for better performance.

Future developments will focus on better electrode-nerve interfaces by improving electrode technology. An increased number of electrical contacts as well as the biological treatment with regeneration of the dendrites growing onto the electrode will increase the number of electrical channels. This will give room for improved speech coding strategies in order to create the bionic ear, i.e. to restore the process of natural hearing by means of technology. The robot-assisted surgery will allow for high precision surgery and reliable hearing preservation. Biological therapies will support the bionic ear. Methods are bio-hybrid electrodes, which are coded by stem cells transplanted into the inner ear to enhance auto-production of neurotrophins. Local drug delivery will focus on suppression of trauma reaction and local regeneration. Gene therapy by nanoparticles will hopefully lead to the preservation of residual hearing in patients being affected by genetic hearing loss. Overall the cochlear implant is a very powerful tool to rehabilitate patients with sensory deafness. More than 1 million of candidates in Germany today could benefit from this high technology auditory implant. Only 50,000 are implanted so far. In the future, the procedure can be done under local anesthesia, will be minimally invasive and straight forward. Hearing preservation will be routine.

## Summary

Cochlear implants are electronical stimulus prostheses for the functional replacement of the inner ear. Because of the rapid technical development and the good results, they could be established as standard therapy for sensory deafness.

Cochlear implantation requires an interdisciplinary team and a quality-controlled concepts that reaches from the indication to the life-long control and that is fixed in the AWMF guideline on cochlear implant [1].

Current cochlear implant systems are partially implantable and equipped with a multitude of additional functions similar to hearing aids for sound pre-processing and noise elimination. The intracochlear position of the electrode allows for a differentiated stimulation of the hearing nerves and thus the transmission of different perceptions of pitches. This simulation of the frequency organization of the inner ear leads to the fact that complex sound signals such as speech are transformed into a differentiated neuronal stimulation pattern of the hearing nerve, which is the base for speech understanding with a cochlear implant.

Today, indications are bilateral sensory hearing loss and deafness in children as well as in adults, single-sided deafness as well as high-frequency hearing loss. Cochlear implants are indicated when a sufficient speech and communication ability (use of the telephone) or speech development are not possible or cannot be expected with alternative methods.

The surgical technique has been standardized and can be applied in all patients. Generally, a transmastoid procedure with posterior tympanostomy and insertion of the electrode through the round window membrane is favored. The fixation of the implant in the bone as well as the secure fixation of the electrode near the cochlear represent crucial elements for a low-complication procedure. Cochlear implant surgery with preservation of hearing is standard today and allows treatment of patients with residual hearing.

Beside the functional control of the implant, the intraoperatively assessed electrophysiological parameters allow an adjustment of the systems, especially in children, based on objective parameters. The subsequent hearing and speech training aims at speech acquisition and speech recognition. The life-long follow-up comprises also technological upgrades and the treatment of complications beside medical and technical controls.

Generally, postlingually deafened patients achieve an open speech understanding and may use the phone. In children, an early implantation after onset of deafness usually leads to a nearly normal speech development.

The complication rate is rather low. Implant failures occur in about 2–4% of the patients, medical complications are observed in about 4% of the implanted people. Re-implantations can usually be performed without any problems. The patients benefit from a technological upgrade. Future developments focus on the bionic ear aiming at the restoration of hearing by simulation of the physiological hearing process by means of the technology. For this purpose, electrodes are developed with a significantly higher number of electrically separated channels. By surface functionalization and additional biological therapies, the regeneration of the hearing nerves with dendrites growing onto the electrode as well as avoiding further degeneration of spiral ganglia cells will be achieved. This may lead to significantly better speech processing strategies that even allow for a tonal hearing, for example of music. Telemedical concepts provide new types of patient care with active involvement of the patients, automated technological implant control, remote care, programming, and technological upgrades. Universal hearing implants will become possible due to multimodal stimulation with integrated intracochlear mechanical or optoacoustic actuators. These implants allow for an individually optimized hearing rehabilitation and can be readjusted at any time in cases of progredient hearing loss. The use of robotic systems will lead to a relevant increase of precision and improved hearing preservation. So-called closed loop systems with measurement of the EEG signal will allow an automated adaptation of the implant system to different hearing situations. Fully implantable hearing systems are currently developed and will make possible the so-called invisible hearing to overcome the stigma of hearing impairment.

## 1 Introduction and basics

### 1.1 Principle of cochlear implant

**Figure 1:**
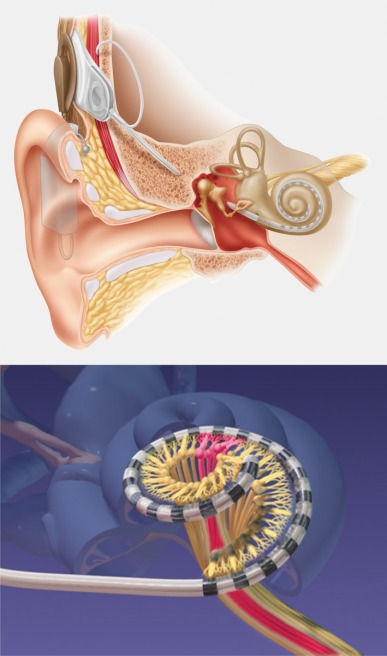
Cochlear implant system, overview (courtesy of Cochlear Company)

**Figure 2:**
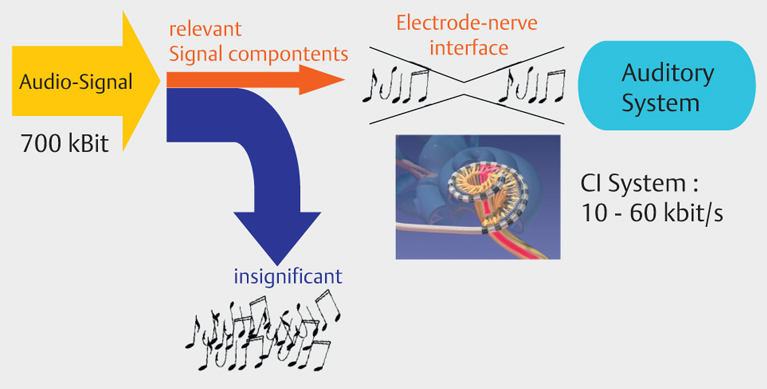
Bottleneck of the electrode-nerve interface (according to A. Büchner)

Cochlear implants are electrical prostheses that trigger auditory sensations via a direct electrical stimulation of the hearing nerve. They replace the function of the inner hair cells that have the role of biological microphone. Hereby, a technical simulation of the natural hearing process is performed with tonotopic presentation of the frequencies along the basilar membrane on different parts of the hearing nerve (Figure 1 (Fig. 1)). In comparison to the natural hearing process, only a low number of electrically separated channels is available for signal transmission. This bottleneck of the electrode-nerve interface becomes especially obvious when listening to music or understanding speech in noise (Figure 2 (Fig. 2)). It is much easier to simulate the time structure of the acoustic signal by high stimulus repetition rate with several 1000 pulses per second per electrode contact.

### 1.2 Historical development

First attempts of hearing rehabilitation were performed by Djourno and Eyries in Paris at the end of the 1950ies [2]. In the following years, other pioneering teams consisting of ENT surgeons and engineers, developed different systems for electro-stimulation of the hearing nerve with intraneural, intracochlear, and extracochlear electrode systems. Already in 1963, Zöllner and Keidel formulated the basic principles of intracochlear multichannel stimulation, which is the base of today’s cochlear implant systems, with up to 20 electrode contacts in the scala tympani for simulation of the tonotopy by use of different stimulus modalities [3]. First clinically applicable systems were developed by House and Urban, later by Hochmair and Hochmair, Clark and Patrick as well as Merzenich in the USA and Chouard in Paris [4].

Even if the initial systems showed a high failure rate, the adaptation of the technology of the cardiac pacemaker led rapidly to a relevant improvement of the reliability of cochlear implants. The transcutaneous transmission substituted soon the percutaneous connector system that was often related to complications. Because of the technical and surgical difficulties despite good channel separation, the intraneural stimulation (Blair Simmons, Zwicker and Leysieffer) was not further developed [5].

### 1.3 Current cochlear implant systems

Current systems insert intracochlear electrodes in the scala tympani, which allows reliable stimulation of the hearing nerve without relevant complications [6].

## 2 Technology

### 2.1 Cochlear implant systems

**Figure 3:**
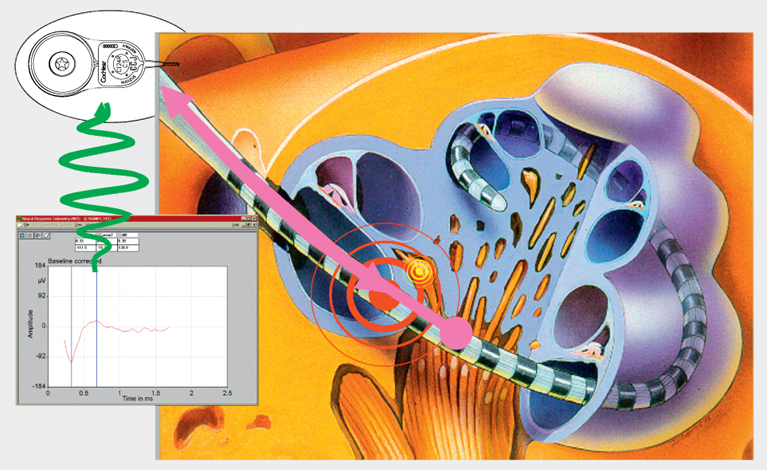
Neural response telemetry (courtesy of Cochlear Company)

The cochlear implant systems of today are generally conceived as two component systems. The external speech processor is used for sound recording, sound pre-processing, and transformation of the acoustic information into a logical sequence of electrical impulses (so-called speech processing) and it is the sender of the FM signal for a transcutaneous transmission and power supply of the implant per induction by a sending coil. The implant that is located under the skin, contains the receiver coil for reception of the FM signal, a demodulator for extraction of the electrical pulses, an electrode carrier with different intracochlear electrode contacts for transmission of the electrical impulses to the hearing nerve and telemetric measurement systems. Current cochlear implant systems dispose of a broad spectrum of signal pre-processing including directional microphones, beam former, noise elimination, and acoustic scene analysis, and of a mostly wireless interaural connection of the systems in cases of bilateral and bimodal treatment. In this way, both speech processors can be synchronized. Telemetric measurement systems record electrophysiological data with the implant itself such as electrode impedances, measurement of acoustically and electrically evoked potentials (Figure 3 (Fig. 3)). Some systems may assess the intracochlear components of electrocochleography with Cochlear Microphonics, compound action potential of the hearing nerve and summation potential. Additionally, electrically triggered stapedius reflexes can be measured. Those objective measurements allow the intra- and postoperative functional control of the implants and provide support for adjustment, which is a great advantage in children. They also allow an indirect control of the position of the implant electrodes.

### 2.2 Electrode systems

**Figure 4:**
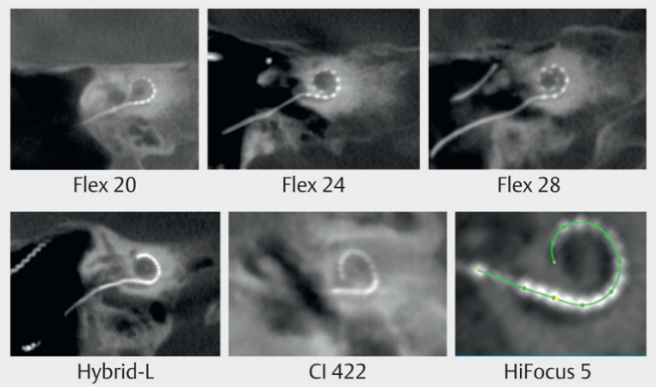
CI electrodes of different lengths

**Figure 5:**
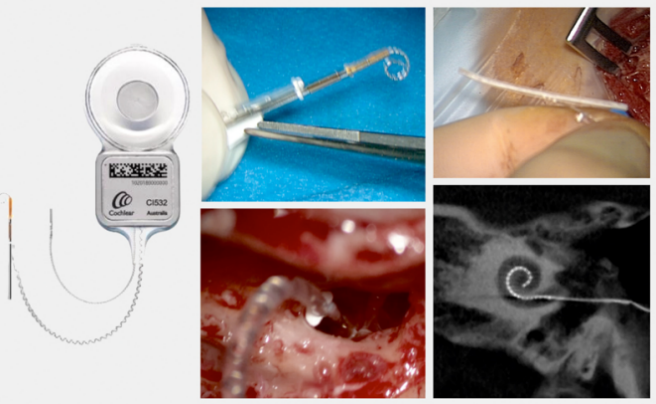
Peri-modiolar electrode

Different electrode systems are available for the individual cochlear implantation (Figure 4 (Fig. 4)). Generally, a sufficient cochlear coverage should be achieved in order to stimulate possibly all spiral ganglia cells. For this purpose, insertion depths of 360° and more are needed. Some manufacturers postulate a higher cochlear coverage to reach apical neuronal elements. For cochlear implant surgery with hearing preservation, specially designed thin electrodes are used that are most frequently placed on the lateral wall and advanced depending on the hearing loss. To achieve a possibly selective stimulation with low stimulation current, preformed, perimodiolar electrodes are inserted, but generally it is less probable to preserve the hearing ability (Figure 5 (Fig. 5)).

Special electrodes are available for malformations and for ossified cochleas (double or split array, Figure 27). Those arrays distribute the electrode contacts on 2 electrode carriers that are inserted into the first and second turns via 2 cochleostomies [7]. Compressed arrays are shortened electrode carriers with a normal number of stimulus contacts that are placed into the drilled initial part of the basal turn. Both procedures aim at approaching the number of intracochlear stimulus contacts as near as possible to the normal cochlear anatomy. To achieve a secure watertight closure of the cochlea in cases of malformations, special electrodes with thickened basal end are applied.

### 2.3 Accessories

The systems are equipped with a broad range of accessories such as for example FM systems, Bluetooth, additional microphone, telephone coil, and connectors for audio-technology. Special protective devices even allow the use in water. Due a special inputs, those accessories are useful to improve speech understanding under unfavorable conditions (classroom, lessons, lectures) as well as the simplified use of the telephone and other communication devices and audio-technology (MP3 player).

### 2.4 Telemedicine 

The possibilities of telemedicine allow the remote fitting, control of the implants (remote care) as well as software upgrades. In the future, also self-fitting of the implant system by the patient himself will be supported.

**Figure 6:**
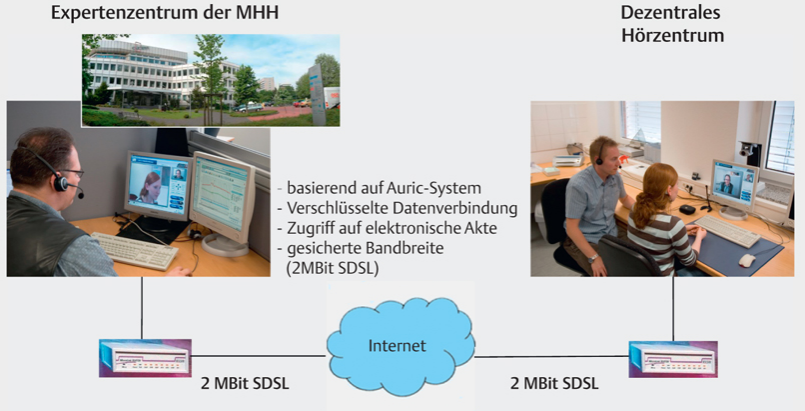
Technical realization of remote fitting (according to A. Büchner). Expert center of the Hannover Medical School. Decentral hearing center, based on the Auric system, coded data connection, secured bandwidth (2 Mbit SDSL)

Patients may connect themselves or in cases of decentral partners via teleprocessing with the cochlear implant center (Figure 6 (Fig. 6)). At any time, the expertise of the center is available (it is no longer necessary to fix appointments that are time consuming and associated with significant secondary expenses). The data of regular and automated measurements of the implant function and the electrode-nerve interface via impedances can be communicated via mobile phones to the center and be evaluated there. Critical variations of the normative values are sent to the implant center and the patient so that medical or technical intervention is rapidly possible. Those variations include for example the increase of the electrode impedances as an indication of a labyrinthitis onset [8].

## 3 Indication and diagnostics

### 3.1 Preconditions 

The indication for cochlear implant is generally given when a sufficient speech understanding and thus ability to communicate or a hearing-oriented language acquisition cannot be achieved by means of alternative hearing rehabilitation methods.

The basic condition for successful application of cochlear implants is a functional hearing nerve and intact central auditory pathways. Furthermore, an anatomically developed cochlear must be present for insertion of the electrode carrier and the connection to the hearing nerve must be intact. The possibility of rehabilitation must be confirmed. A sufficient cognitive competence is relevant for the hearing-speech training and also for the hearing and language acquisition as well as speech understanding. Limitations of the cognitive performance such as for example dementia, are a negative prognostic factor [9].

Additional disabilities must be identified early, especially in children, with regard to their impact on hearing and language acquisition and taken into consideration for therapy planning.

Today, the following general indications are applied for cochlear implantation:

- Bilateral high-grade hearing impairment or sensory hearing loss near to deafness
- Unilateral sensory deafness
- High frequency hearing loss with residual hearing in the low frequencies.

In cases of unilateral deafness, the bilateral partly also binaural hearing should be restored. The use of additional tinnitus suppression is possible [10], [11].

The preservation of the residual hearing ability in the context of high-frequency hearing loss allows the use of electroacoustic stimulation. In the future, new treatment concepts for presbyacusis might be developed [12], [13], [14].

### 3.2 Diagnostics

**Figure 7:**
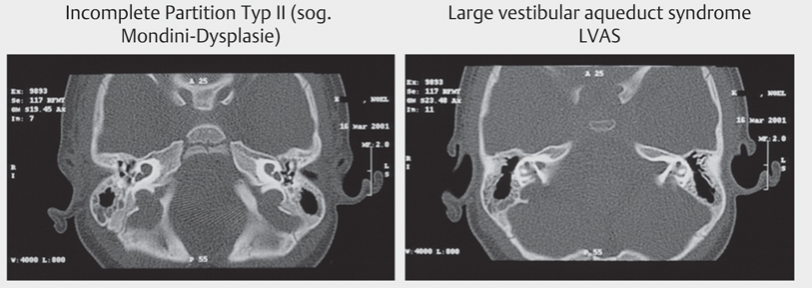
Cone beam tomography of the temporal bone

**Figure 8:**
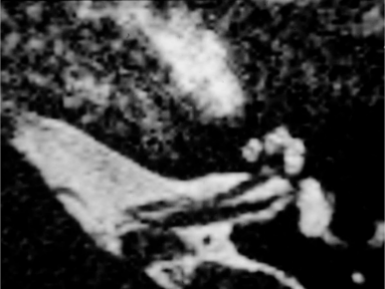
MRI of the temporal bone, T2 image of the cochlea and the internal auditory meatus

The indication requires standardized diagnostics that beside general age-independent examinations include additional elements for adult and children (Table 1 (Tab. 1), Figure 7 (Fig. 7), Figure 8 (Fig. 8)).

**Figure 9:**
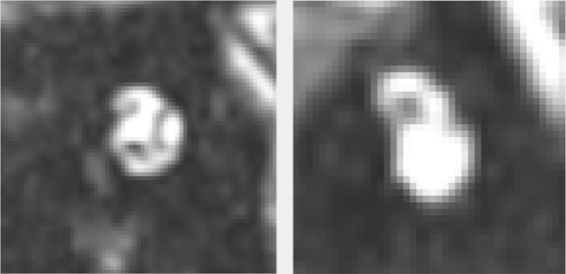
MRI, T2 image of a transversal section through the internal auditory meatus. Aplasia of the hearing nerve on the right side (right image) and normal findings on the left with acoustic nerve (left below), facial nerve (left above), and vestibular nerve (right)

Because of the small dimensions of the structures, high resolution imaging, especially of neuronal structures, is most important. Since often malformations are the cause of pediatric deafness, an exact assessment and description is essential for the therapeutic approach. The verification of inner ear structures requires special sequences and section planes so that for example all 4 nerves may be identified in the internal auditory meatus (facial nerve, superior vestibular nerve, inferior vestibular nerve, acoustic nerve) (Figure 9 (Fig. 9)).

**Residual hearing:** It is very important to assess the hearing ability in order to estimate the benefit for speech understanding as well as the potential for hearing and language development. While the focus is placed on psychoacoustic procedures in adults, the diagnostics in children are supported by objective measurements. If required, also follow-up examinations are indicated, for example in cases of substantial residual hearing and progredient hearing loss (see chapter on limiting cases).

#### 3.2.1 Functional test of the hearing nerve and the central hearing pathway

Generally, the function of the hearing nerve may be verified with the so-called promontory test. By means of a needle electrode that is either placed transtympanically on the promontory or by means of auditory canal electrodes, stimulation currents of different frequencies between 50 and 1600 Hz may be applied that trigger an auditory sensation in the patient. With decreasing frequencies, the dynamic range narrows due to then dominating pain sensation. Auditory fatigue or a missing hearing impression may indicate a neural or central deafness. An objective functional test is possible by recording electrically evoked brainstem potentials. Despite the low sensitivity, this method is a valuable diagnostic procedure for pediatric patients. Further objective procedures are methods of functional imaging with identification of an increased activity in the area of the auditory cortex under electrical stimulation. Those are for example the positron emission tomography (PET) [15], functional magnetic resonance imaging (fMRI) [16], and near-infrared spectroscopy (NIRS) [17]. Except magnetic resonance imaging, both other procedures can also be applied for postoperative functional testing of the hearing pathway. Hereby, NIRS has the advantage that it may be repeated frequently since there is no radioactive exposure. It may also be applied for follow-up examinations to assess the developmental status of the hearing pathways and therapy induced plasticity processes for example after cochlear implantation.

#### 3.2.2 Individual anatomy of the cochlea

CT scan and CBT further allow sizing of the cochlea, especially of its length. This is the basis for selection of the electrode carrier and for determination of the insertion depth in the context of individualized cochlear implantation [18].

#### 3.2.3 Genetic diagnostics

One relevant pillar of the etiological clarification is the genetic diagnosis. About 50–60% of pediatric hearing impairment are due to a genetic predisposition. They are classified into non-syndromic and syndromic types of hearing loss, i.e. generally the hearing loss is a symptom in the context of a syndrome.

Currently, more than 100 so-called deafness genes are known. The most frequent expression is the mutation on the gene locus GJB2 that leads to a disorder of the connexin molecule (Connexin 26), a gap junction protein. The consequences are disorders of the ionic homeostasis of the hair cells that are irreversibly damaged by a potassium intoxication [19], [20].

The difference is made between autosomal recessive, autosomal dominant, X-linked, and mitochondrial, genetically caused hearing losses. Autosomal recessive types often reveal deafness already at birth and are entitled DFNB (e.g. DFNB1 with connexin 26 disorders). Autosomal dominant types often have a postnatal onset and are progredient (DFNA). X-linked hearing losses are located on the X chromosome and only appear in males (DFN). Mitochondrial hereditary hearing impairment is passed on by maternal genes.

### 3.3 Indications

#### 3.3.1 Indications in adults

Generally, the patients who deafened postlingually, dispose of an acoustic memory. They benefit highly from cochlear implantation. The important inter-individual variability reveals further prognostic factors. Those are the duration and the time course of deafening, the presence and the use of residual hearing by hearing aids as well as cognitive abilities and their impairment, for example in the context of pre-dementia (so-called cognitive decline) [21].

The following audiometric limit values are applied today for adults:

1. Mean hearing threshold in the audiogram (250–4000 Hz) >75 dB HL
2. Speech understanding in the Freiburg monosyllabic test <45% at 65 dB under best-aided condition (hearing aids)

The last mentioned value is based on the ability to use the telephone. Phone calls are possible with an understanding of monosyllables >50%.

The measurement of speech understanding in noise provides an additional criterion for the evaluation of communication disorders under difficult acoustic conditions (e.g. HSM sentence test, OLSA, HINT, AzBio) [22], [23].

#### 3.3.2 Indications in children

The origins of deafness in children are manifold. The difference must be made between congenital and acquired causes as well as the time of deafness (pre-, peri-, or postlingual). If the onset is observed before language acquisition, the term of pre-lingual deafness is applied, after final language acquisition (around the 10^th^ year of life) it is called post-lingual deafness. If hearing loss is detected during the phase of language acquisition, it is the case of a peri-lingual deafness.

The impact of hearing loss on the language development is well-known. It is crucial for the therapeutic success of a cochlea implant to possibly early detect, diagnose, and treat hearing loss in order to keep the consequences of the auditory deprivation for hearing and language development as well as the general mental development on a low level [24]. Hereby, also the development of a binaural hearing system as base of directional hearing and speech understanding in noise must be mentioned.

In cases of congenital deafness, the newborn hearing screening is essential for early detection. Due to the widespread provision, the identification of children with uni- or bilateral hearing loss is possible immediately after birth [25], [26]. If hearing loss appears during the first years of life, especially the application of objective audiometric measurements is indicated for early detection and follow-up beside observation by the parents.

If a child is suspected to suffer from hearing loss, confirmation diagnostics have to be performed immediately by means of standardized diagnostic programs (Table 1 (Tab. 1)). Those diagnostic procedures allow determining the extent and type of hearing loss and the necessary therapy may be introduced.

The indication for a cochlea implant in children is given when no defined stimulus responses may be obtained by objective measurements. According to the current standard, objectively determined thresholds of less than 80 dB are a clear indication for cochlear implant if normal hearing or language development cannot be expected with alternative therapeutic options (hearing aids).

The maturation processes in the field of the hearing system have to be observed. Initially, slightly increased hearing thresholds may improve or normalize in the context of maturation. In cases of higher-grade sensorineural hearing loss, however, this cannot be observed [27].

Cochlear implantation in children should be performed immediately after indication. Generally, it may be done after the 6th month of life in the first year. Bilateral implantation should be performed in cases of bilateral deafness, if possible simultaneously or otherwise as sequential implantation with a short time interval in order to use the sensitive phase for the development of binaural hearing [28], [29].

Hearing aid fitting beside unilateral cochlear implantation is indicated in cases of asymmetric hearing with unilateral sensory hearing loss and useable hearing ability in the contralateral ear. Narrow controls of the residual hearing as well as the hearing success in this ear must be performed so that progredient hearing loss or development failure of bilateral hearing are detected in time and the point of then necessary sequential cochlear implantation of the second ear may be correctly chosen [28], [30], [31].

If maturation is delayed, as it may be expected in cases of increased hearing thresholds and prolonged inter-peak latencies in brainstem audiometry, control examinations in narrow intervals and probatory hearing aid fitting seems to be appropriate.

Often hearing improvement is observed due to maturation of the peripheral auditory system and the central hearing pathway so that a repetition of the examination of the residual hearing after some weeks or months is indicated. If no significant improvement of hearing is observed, cochlear implantation should be performed in the second year of life at the latest.

In this context, the so-called perisynaptic audiopathy must be mentioned summarizing disorders of the inner hair cells, the synapsis to the afferent nerve fibers as well as true auditory neuropathy with damage of the afferent neuron (auditory neuropathy spectrum disorder). This term encompasses different pathophysiological disorders of stimulus transmission and stimulus forwarding in the peripheral auditory system. Only in the context of true auditory neuropathy a relative contraindication for a cochlear implant system exists.

The typical constellation of the findings is described in Table 2 (Tab. 2) [32].

In cases of particular urgency such as for example threatening cochlear obliteration by labyrinthitis and meningitis require immediate, early implantation.

#### 3.3.3 Limiting cases

Limiting cases describe patients with a relatively good hearing and a comparable poor speech understanding, e.g. often in cases of Menière’s disease, fluctuating hearing ability, or combined hearing loss with a bone conduction threshold that is difficult to determine. In such cases, the exact differential diagnosis is relevant with application of objective measurement procedures including electrocochleography. If after optimized hearing aid fitting and sufficient observation no improvement of speech understanding is observed, cochlear implantation is indicated.

If malformations or obliterations lead to a missing hearing and language development in children or missing hearing impression in post-lingually deafened patients, a central-auditory implant, generally an auditory brainstem implant (ABI), is indicated in cases of bilateral disorder [15]. This also applies for patient with condition after removal of an acoustic neuroma, fractures of the temporal bone, or damage of the hearing nerve as well as auditory neuropathy. It depends on the individual case (neural or central deafness), if first probatory CI has to be performed.

## 4 Surgical technique and implantation

### 4.1 Standard surgical technique

**Figure 10:**
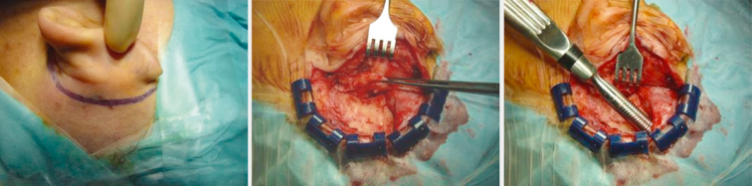
Retroauricular incision. Periostal flap. Periostal pouch.

**Figure 11:**
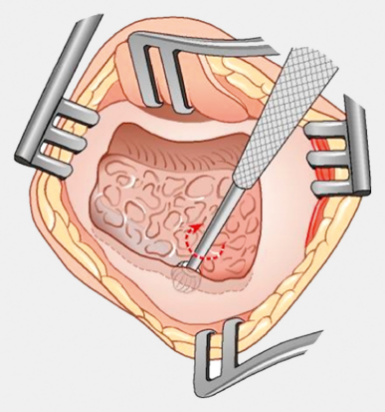
Mastoidectomy with cortical projection (courtesy of Endo-Press, Tuttlingen, Germany)

**Figure 12:**
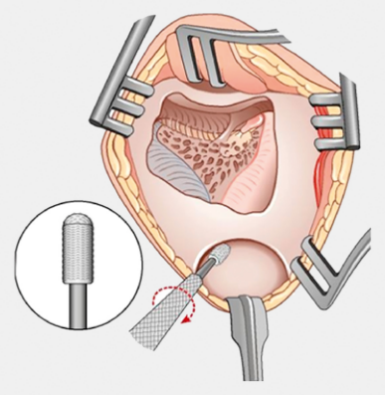
Creation of the bone bed (courtesy of Endo-Press, Tuttlingen, Germany)

**Figure 13:**
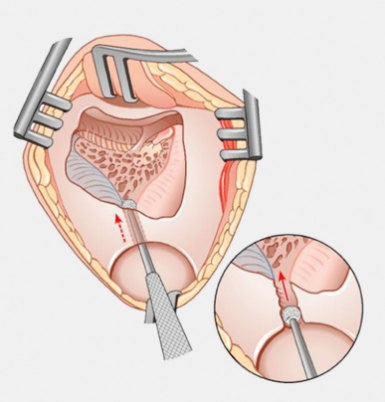
Creation of a connecting tunnel/canal from the bone bed to the mastoid (courtesy of Endo-Press, Tuttlingen, Germany)

**Figure 14:**
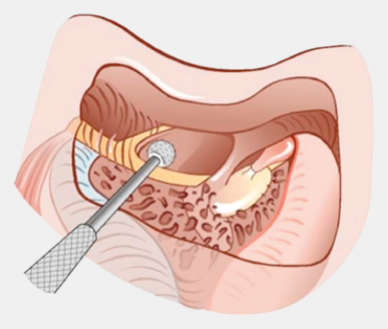
Posterior tympanostomy (courtesy of Endo-Press, Tuttlingen, Germany)

**Figure 15:**
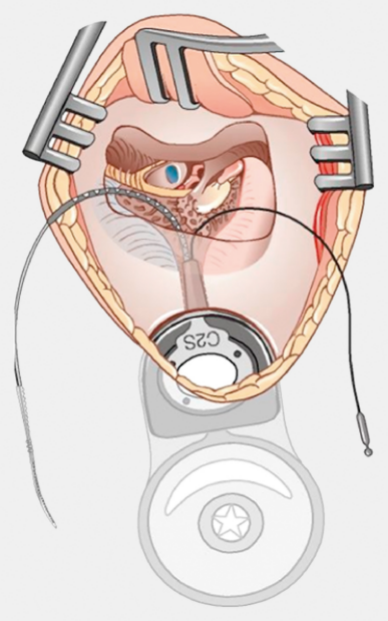
Insertion of the implant (courtesy of Endo-Press, Tuttlingen, Germany)

**Figure 16:**
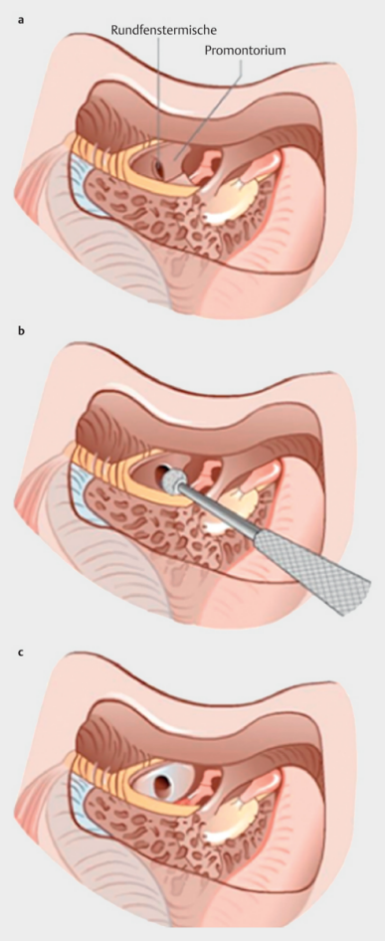
Exposition of the round window membrane (courtesy of Endo-Press, Tuttlingen, Germany)

**Figure 17:**
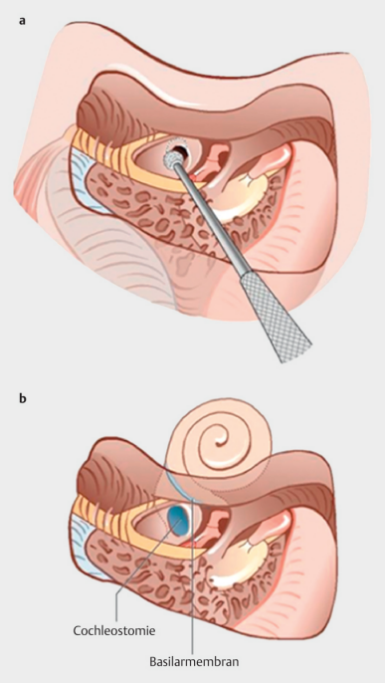
Cochleostomy (courtesy of Endo-Press, Tuttlingen, Germany)

**Figure 18:**
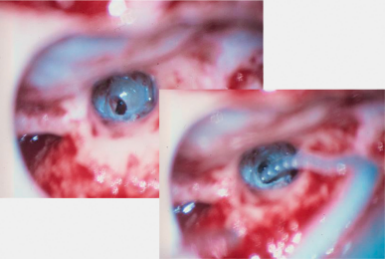
Insertion of the electrode through the round window

**Figure 19:**
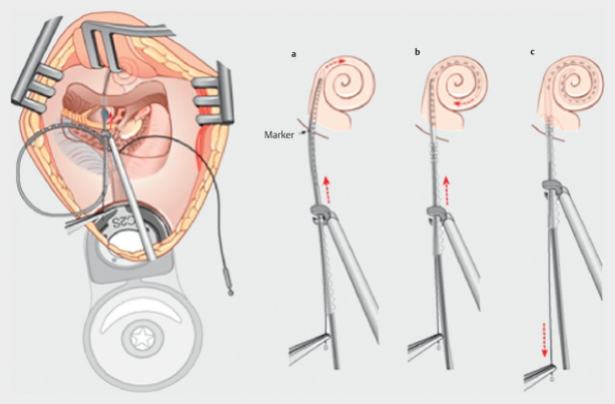
Electrode insertion by so-called advanced-off stylet technique (courtesy of Endo-Press, Tuttlingen, Germany)

**Figure 20:**
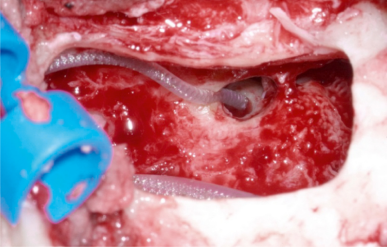
Fixation of the electrode in a bone slit in the posterior tympanostomy

**Figure 21:**
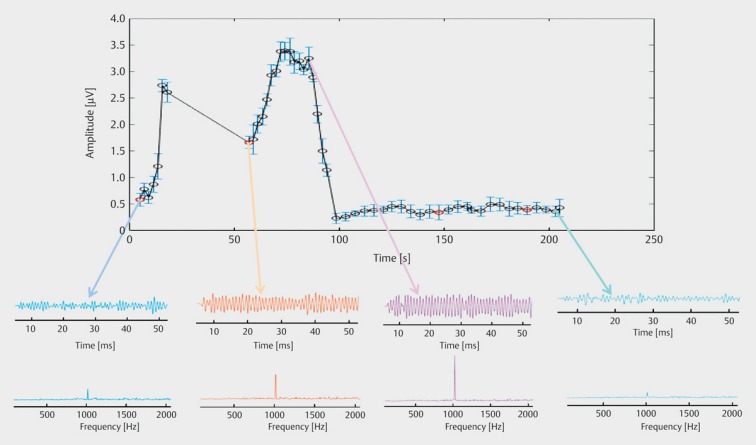
Intraoperative cochlear monitoring. Registration of Cochlear Microphonics. Amplitude decline during insertion of the electrode in a case of cochlear damage.

The surgical technique is largely standardized and may be applied in all age groups and special cases. Generally a transmastoid surgical approach with posterior tympanostomy is performed comprising the following steps [33]:

1. Retroauricular incision (Figure 10 (Fig. 10)).
2. Creation of a periostal pouch in occipital direction to insert the receiving part of the implant (Figure 10 (Fig. 10)).
3. Partial mastoidectomy with exposure of the posterior wall of the auditory canal, the antrum with the incus, the mastoid course of the facial nerve, and the canal of the chorda tympani, the labyrinthine block with the 3 semicircular canals, the sigmoid sinus, and the cortex to the middle and posterior cranial fossa as well as the sinus-dura angle (Figure 11 (Fig. 11)).
4. Creation of a bone bed to insert the implant at 1 cm behind and above the sinus-dura angle. Hereby, advancing sometimes onto the dura is necessary, especially in children. Afterwards careful coagulation is performed (Figure 12 (Fig. 12)).
5. Creation of a connecting canal or tunnel to the mastoid in projection on the sinus-dura angle to securely insert and fix the electrode (Figure 13 (Fig. 13)).
6. Performance of the posterior tympanostomy by removal of the bone between the bone-covered facial nerve and the chorda tympani (Figure 14 (Fig. 14)). If necessary, the chorda tympani has to be removed, displaced and newly embedded (neurolysis) to achieve a sufficient approach to the middle ear and the relevant structures of the inner ear. Those are the promontory, the round and oval windows with stapes and stapedius tendon. In children, revisions, and malformations generally intraoperative monitoring of the facial nerve should be performed to avoid neural damages and facilitate identification of the nerve.
7. Insertion of the implant and positioning of the electrodes (Figure 15 (Fig. 15)).
8. Preparation of the round window membrane with removal of the bone, if needed, to completely visualize the membrane (Figure 16 (Fig. 16)).
9. Opening of the cochlea (Figure 17 (Fig. 17)) by incision of the round window membrane or cochleostomy.
10. Insertion of the electrode carrier (Figure 18 (Fig. 18) and Figure 19 (Fig. 19)). Generally this should be performed in an atraumatic and slow procedure. The selected insertion depths depend on the size of the cochlea (individual sizing by means of CBT [18]) as well as the dimension of residual hearing. The insertion is performed down to the calculated depth.
  Depending on the electrode type, different insertion techniques, possibly using special instruments, are required. Lateral wall electrodes may be easily inserted by means of a specially developed insertion forceps in one-hand technique (Figure 18 (Fig. 18)).
  Preformed electrodes require special insertion techniques. In the context of advanced-off stylet technique, the electrode is advanced into the cochlea by the stylet after partial insertion (Figure 19 (Fig. 19)). For another electrode type, the electrode is slowly advanced through an insertion tube and afterwards the tube is removed from the cochlea (Figure 5 (Fig. 5)).
11. Closure of the cochlea. To avoid perilymph fistula, a secure closure of the cochlea is essential. Either a fascia collar may be used that had been created before insertion, or muscle pieces that are positioned carefully around the electrode opening.
12. Positioning of the electrode carrier. The electrode carrier has to be securely positioned in the mastoid in order to avoid the contact with the covering skin. Furthermore, it is important to fix the electrode carrier near the cochlea to avoid migration in direction of the mastoid. Hereby, different techniques may be applied:
  - Bone slit at the inferior edge of the posterior tympanostomy to clip the electrode (Figure 20 (Fig. 20)).
  - Use of a clip for fixation at the bridge.
  - Use of different gluing materials such as bone cement or fibrin glue.
  - Use of a special rotation tube that is clipped onto the electrode
13. Intraoperative electrophysiology is obligatory for the intraoperative functional control of the implant as well as for measuring the stimulus response of the nerve. Via an attached coil system the implant can be activated. Then the following measurements can be performed:
  - Electrode impedances
  - Electrically triggered stapedius reflex with determination of the threshold
  - Electrically triggered compound action potential of the hearing nerve (NRT, neural response telemetry) (Figure 3 (Fig. 3))
  - Cochlear monitoring
  During insertion of the electrode, residual hearing can be monitored by measuring cochlear microphone potentials outside and inside the cochlea. Critical changes of the Cochlear Microphonics amplitude indicate an impairment of the inner ear function. By repositioning the electrode, a permanent hearing loss might be avoided [34], [35], [36] (Figure 21 (Fig. 21)).
14. Appropriate wound closure in several layers to securely cover the implant.
15. The intraoperative control of the electrode position by radiography or CBT is the standard in order to identify insertion failures in time and to correct them in the same session (Figure 4 (Fig. 4)). Furthermore, the insertion depth has to be critically verified and to be corrected, if necessary. Radiography provides important information for the postoperative fitting [36]. The chosen surgical technique is associated with a very low complication rate.

#### 4.1.1 Implantation in children

Because of the immaturity of the organs, the implantation should be performed generally from the 6^th^ month of age within the first year of life in cases of congenital deafness. Only in cases of particular urgency such as the risk of obliteration in the context of labyrinthitis, the implantation should be performed earlier. The implantation should include both sides to allow the development of binaural hearing with the ability of directional hearing and improved speech understanding in noise. Hereby, the simultaneous implantation should be preferred if it is possible from an anesthesiologic point of view. Compared to sequential implantation, the following advantages should be mentioned:

- Only one hospital stay
- Only one anesthesia for surgery
- Simultaneous activation of the hearing system for the development of binaural hearing

**Figure 22:**
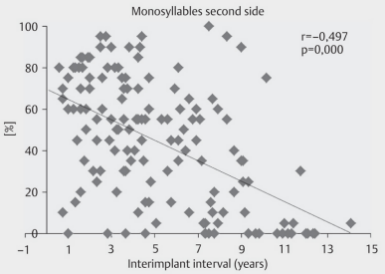
Bilateral sequential implantation. Hearing performance of the 2nd side depending on the inter-implant interval (according to Illg et al., 2013 [28]).

**Figure 23:**
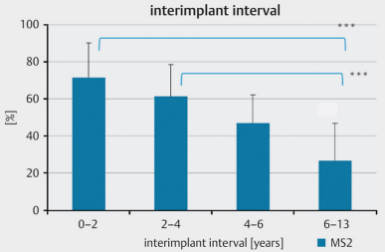
Bilateral sequential implantation: Understanding of monosyllables of the 2nd ear compared to the 1st ear depending on the inter-implant interval (according to Illg et al., 2013 [28]).

The disadvantage is the prolonged anesthesia and the duration of surgery with the associated risk for example of increased blood loss. If sequential bilateral cochlear implantation is performed, the interval should be rather short in order to keep the auditive deprivation of the second ear as low as possible and thus to achieve a similar hearing ability for both ears. If the intervals are longer, poorer hearing results in the later implanted ear and poorer bilateral and binaural hearing performance are observed (Figure 22 (Fig. 22) and Figure 23 (Fig. 23)) [28].

### 4.2 Alternative surgical procedures

Alternative approaches have been described such as the suprameatal technique. The posterior tympanostomy is not performed and the electrode is advanced between the auditory meatus and the incus in direction of the middle ear. The comparably narrow access as well as the unfavorable angle for an atraumatic insertion of the electrode are critical restrictions of this procedure, especially in the context of hearing preservation [37].

### 4.3 Robotic or minimally invasive surgery

**Figure 24:**
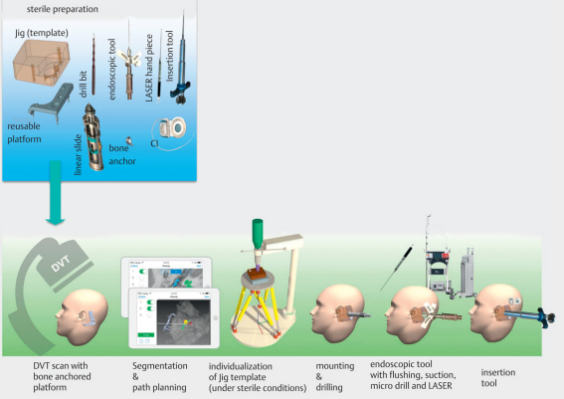
Robofig. Workflow of the robotic minimally invasive cochlear implantation

In the future, minimally-invasive procedures will gain in importance. By the application of robotic systems, ideal trajectories may be determined preoperatively, along which for example drilling is performed from the mastoid surface to the cochlea [38]. The cochlea can be opened through this drill canal and the electrode is securely inserted by means of special insertion tools (Figure 24 (Fig. 24)). Advantages might be a shorter duration of surgery, reduced insertion trauma as well as an exact intracochlear positioning of the electrode taking into account the individual anatomy [39].

### 4.4 Special cases

Special cases occur when the surgical standard procedure has to be modified. Those cases are:

- a) Hearing preservation
- b) Obliteration of the cochlea
- c) Malformations
- d) Re-implantations

Those cases require special surgical techniques, large experience of the surgeon, special equipment and special implants.

#### 4.4.1 Cochlea implant surgery and hearing preservation

**Figure 25:**
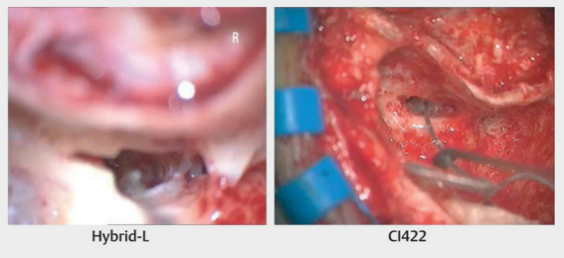
CI surgery with hearing preservation. Insertion through the round window.

**Figure 26:**
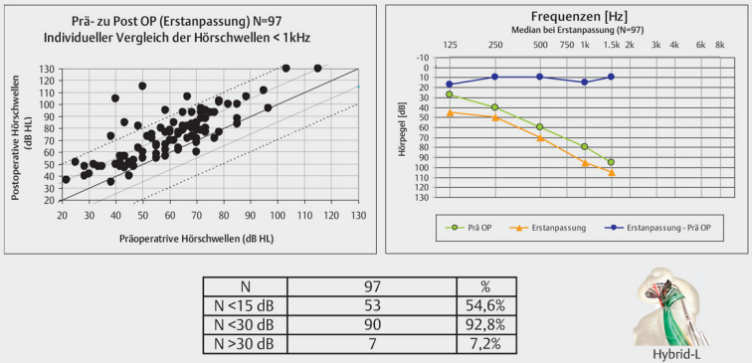
Cochlear implantation with hearing preservation – comparison of pre- and postoperative hearing thresholds for hybrid L electrode – difference and percentage of good (<15 dB), any (<30 dB) hearing preservation as well as deafness rate

To preserve residual hearing, a so-called soft-surgery technique has to be applied. Generally, the insertion is performed through the round window membrane. The electrode is carefully inserted to a pre-determined depth and then fixed (Figure 25 (Fig. 25)). Suction and drilling dust have to be avoided imperatively. Generally, good hearing preservation outcomes may be achieved reliably (Figure 26 (Fig. 26)) [14]. The outcome clearly depends on the length of the electrode as well as the size of the individual cochlea. Insertion depths of less than 18 mm show significantly better hearing preservation than higher insertion depths [40]. The use of local or systemic steroids may also have a protective effect. As mechanisms for postoperative hearing loss, different aspects are discussed such as for example foreign body reaction regarding the implant, mechanical damage of intracochlear structures, or functional impairment of the cochlear mechanics [41].

#### 4.4.2 Obliteration of the cochlea

The obliteration may be due to connective tissue or bone and develops generally after labyrinthitis or extended otosclerosis or trauma with transverse fracture. The difference must be made between partial and total obliterations. In the context of partial obliteration, generally the initial part of the basal turn is affected. Appropriate drill-out techniques allow removing the newly developed tissue and reaching the open part of the scala tympani. Usually, the electrode can then be inserted according to the standard procedure. Alternatively, also an access to the scala vestibuli can be used that is reached by cochleostomy in front of and above the round window.

**Figure 27:**
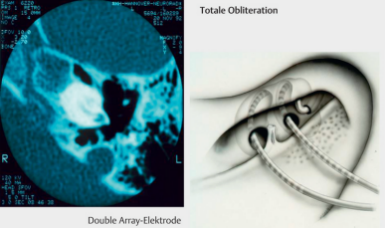
Obliteration of the cochlea (taken from Lenarz et al., 2001 [7])

Total obliterations require the application of so-called drill-out techniques. Besides drilling out the basal turn, a second cochleostomy is performed via the round window below the cochleariform process (Figure 27 (Fig. 27)). In this way, either the ascending part of the basal turn is reached or, if the drilling is performed more in cranial direction, the scala tympani of the second turn. This allows insertion of so-called double or split arrays with 2 electrode carriers to achieve a possibly high number or intracochlear stimulus contacts.

#### 4.4.3 Malformations

Malformations may have very different appearances. They reach from complete aplasia via hypoplasia to incomplete partition of the cochlea. Furthermore, different malformations of the hearing nerve are observed with finally even aplasia (Table 3 (Tab. 3)) [42].

The hearing results are manifold and depend from the anatomical situations. The crucial factor is the status of the hearing nerve and the presence or absence of tonotopic organization of the cochlea. Often, a syndromic hearing loss is observed so that additional disabilities may co-influence the outcome [43].

**Figure 28:**
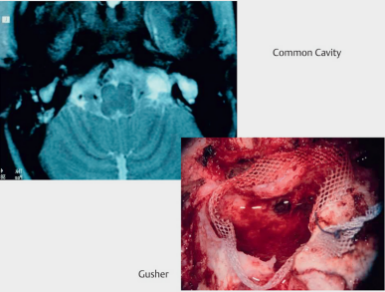
CI in cases of malformations. Common cavity.

**Figure 29:**
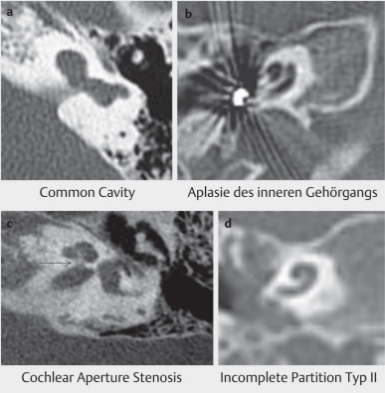
Malformations of the temporal bone. a common cavity; b aplasia of the internal auditory meatus; c cochlear aperture stenosis; d incomplete partition type II (see text and Table 3).

Frequently, abnormally wide connections to the internal auditory canal are found with the consecutive risk of a gusher during implantation (Figure 28 (Fig. 28)). Additional malformations as for example arachnoid cysts of the footplate must be identified. The cochlear aperture stenosis is characterized by narrowing of the cribriform lamina of the hearing nerve at the transition of the internal auditory meatus to the inner ear with consecutive hypoplasia or aplasia of the hearing nerve (Figure 29c (Fig. 29)).

Implantation requires a procedure that takes into account the anatomical situation and it should be performed under monitoring of the facial nerve. Intraoperatively, radiographic control of the electrode position is recommended to identify and correct false positioning (Figure 4 (Fig. 4) and Figure 5 (Fig. 5)). Electrophysiological measurements including EBERA and NRT allow the immediate functional control of the hearing nerve. In the context of IPT I, lateral wall electrodes with ring contacts should be used because the parts of the hearing nerve are located at the posterior wall, similar to the common cavity.

Opening of the cochlea should be performed carefully only to the extent that corresponds to the electrode diameter. In this way, generally a gusher can be stopped already by electrode insertion.

#### 4.4.4 Re-implantation

The reasons for re-implantation are:

- Device failure
- Medical complications
- Technological upgrade

Generally, re-implantations may be performed without any difficulties. When the electrode is not ingrown, it can be removed and replaced by an electrode of equal or similar structure. In single cases, however, also new bone formations are observed that complicate the extraction of the electrode. In those cases, a procedure comparable to obliteration treatment is required. If electrodes with larger diameters are applied, the complete insertion is sometimes difficult. Hereby, the use of rigid electrode dummies for bougienage of the cochlea seems to be suitable and often successful. If the same implant is used and re-implantation can be performed without complications, the hearing results after surgery are comparable. In cases of technological upgrade with re-implantation of a more modern implant, even better hearing results may be achieved. However, if the electrode is not completely inserted, the hearing results may also be poorer [44].

Medical reasons for re-implantation are usually complications such as implant infection and extrusions or migration of the electrode that may occur in 6–9% of the cases. In the context of infections, generally a 2-step procedure with explantation and later re-implantation is indicated if conservative treatment is not successful. Regarding extra-cochlear infection, the electrode may sometimes remain in situ in the first step [45], [46].

**Figure 30:**
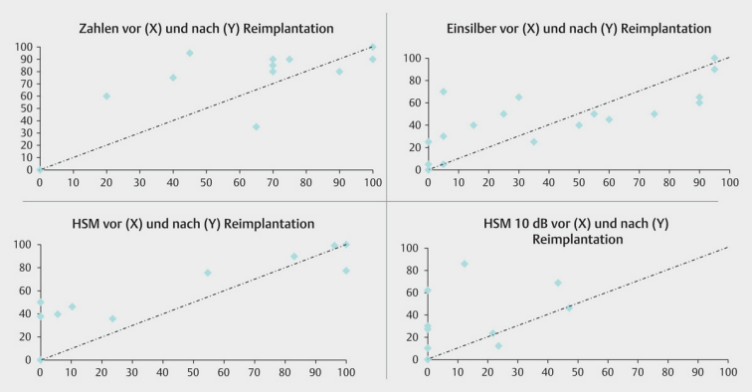
Hearing results before and after re-implantation (first implant: Nucleus 22)

Re-implantations with the purpose of a technological upgrade are controversially discussed. They are basically possible in patients with a technologically outdated implant, which may be the reason for poor hearing. Modern technology can lead to a better performance, however, the advantages must outweigh the risks of re-implantation (Figure 30 (Fig. 30)).

#### 4.4.5 Chronic otitis media

The different types of chronic otitis media require special therapeutic concepts. The following scenarios have to be differentiated:

- Serous or mucous otitis media, so-called otitis media with effusion or sero-/muco-tympanum
- Otitis media chronica mesotympanalis
- Otitis media chronica epitympanalis
- Condition after radical ear surgery

The procedure in the context of sero-muco-tympanum, so-called serous or mucous otitis media, is controversially discussed. Since it is generally the case of bacterial middle ear effusions, first the treatment with paracentesis, drainage and even adenotomy may be initiated [47].

If the treatment is not successful soon, the absence of microbes allows cochlear implantation even when the middle ear is not completely healed, to avoid any delay of the auditive rehabilitation.

In cases of otitis media chronica mesotympanalis, first remediating ear surgery should be performed based on the principles of tympanoplasty. After complete healing of the chronic inflammation, cochlear implantation may be performed in the usual way. The decisive factor is a good middle ear ventilation to avoid retractions of the tympanic membrane and consecutive cholesteatoma development.

In the context of otitis media chronica epitympanalis, first remediation of the cholesteatoma must be performed and – depending on the extent of the findings – cochlear implantation is made in the same session or in the interval.

If a radical cavity is found or if implantations are not possible because of narrow anatomical conditions without preserving the covering bone of the external auditory meatus on the electrode, a procedure in several steps should be chosen. First, the so-called subtotal petrosectomy with closure of the auditory canal is performed; after 6 months and complete healing the actual cochlear implantation takes place. Usually, the inflammation process is eradicated with the result of inflammation-free local circumstances so that an implant loss due to infection or inflammatory reactions can be avoided in most of the cases. A unilateral procedure is only recommended for absolutely inconspicuous and inflammation-free radical cavities [48], [49].

## 5 Postoperative fitting and hearing-speech training

### 5.1 Principles and contents

In adults, the fitting is performed psycho-acoustically with assessment of the co-called T and C levels (threshold and current of comfortable loudness) for every single electrode contact. Then the loudness between the contacts is balanced, the dynamic range is defined, and the speech processing strategy is selected. The strategy is an algorithm according to which the acoustic signal is transformed – completely or partially – into a defined sequence of electrical pulses that are then transmitted to the hearing nerve via the electrode. The aim is a possibly physiological activation pattern of the hearing nerve.

**Figure 31:**
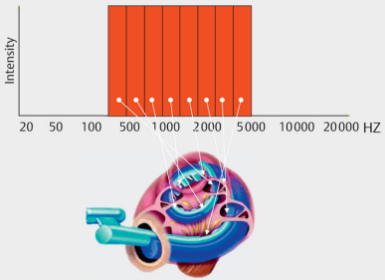
Speech processing. Tonotopic allotting of frequency bands to single electrode contacts (Advanced Bionics Company).

The single electrode contacts are allotted to different frequency ranges in the form of frequency bands (Figure 31 (Fig. 31)). The allocation is made according to the subjective hearing impression and should follow the tonotopic order, i.e. high frequencies should be transmitted to the basal electrodes, low frequencies to the apical electrode contacts. An anatomically correct depictions is generally not possible because the presented frequencies and the position of the electrode contact are usually not congruent with the physiological representation on the cochlea (so-called Greenwood function). In an intraoperative process, an optimized fitting may be achieved until the patient reaches an open speech understanding. After longer intervals of hearing habituation, further optimizations may follow. The time of fitting can be chosen immediately after surgery as early fitting or as first fitting about 4–5 weeks after surgery. Generally the last-mentioned procedure is preferred because then postoperative swellings have disappeared and the intracochlear healing is completed. 

Already during fitting, a hearing and speech training take place. First, the focus is placed on the recognition of basic auditive categories such as loud, quiet/soft, high, low, the recognition of single syllables, of vowels and consonants, later it is speech understanding [50].

In children, fitting is performed based on objectively assessed parameters (see also chapter 2.4 and 4.1). So-called NRT based maps allow an approximation of the profile over the complete electrode carrier as soon as a T and C level can be psycho-acoustically determined on an electrode contact. Additionally, electrically evoked brainstem potentials and EEG signals are applied [51]. In order to control the usage by means of a so-called data-logging, the implant registers several parameters such as the daily time of usage. This information can be used to support rehabilitation [52]. 

### 5.2 Telemedicine (remote care)

Telemedical procedures allow remote fitting. Hereby, the patient is connected with an interacting center via a data transmission line. The specialist in the cochlear implant center is able to observe the patient and talk to him directly. A direct access to the implant is possible by a specialist on site or via an interface that the patient may control himself. In this way, fine tuning, especially with regard to the domestic setting, technology checks, and upgrades of the software may be performed.

Via the telemedical connection, daily implant controls are possible. This allows early detection of increased impedances as sign of a labyrinthitis onset. Remote care is particularly important for the life-long follow-up [53].

### 5.3 Self-fitting

Self-fitting by the patient will also be possible in the future. The patient will be enabled to optimize single parameter settings due to his subjective hearing experience by applying certain interactive procedures, as soon as the first fitting is complete. This procedure will be increasingly used after first fitting and hearing experience.

## 6 Rehabilitation and follow-up

### 6.1 Caring models

To support hearing and speech acquisition, especially in children, specific rehabilitation measures are suitable. This is also true for adults who experience only slow progress or who benefit from a more intense therapeutic approach because of unfavorable prognostic factors such as long-term deafness. Significantly better hearing results may be achieved in this way [54].

### 6.2 Life-long follow-up

After implantation, the surgeon is responsible to organize and perform a life-long follow-up. It refers to the technical check-up as well as the settings of the implants. Furthermore, regular updates of the software and hardware are necessary since they make progress of the implant technology useable for the patients. Besides, medical complications and functional failure can be detected and addressed.

### 6.3 Remote care

See chapter 5.2.

## 7 Results

### 7.1 Test procedures for assessment of the hearing performance

The outcome is assessed and documented by means of standardized test procedures.

The thresholds with cochlear implant are measured. They should amount to values between 20 dB and 30 dB over the whole frequency spectrum. The consistent amplification over all electrode contacts is important for a good hearing result.

For the assessment of speech understanding, age-dependent test procedures are available. They register the speech understanding for numbers and monosyllables as well as the understanding of sentences in quiet and in defined noise.

Generally the Freiburg speech intelligibility test for monosyllables is used in adults. Comparative evaluations regarding the preoperative hearing status as well as the development of the speech understanding over the time may be documented. In addition, speech tests in noise such as the Oldenburg sentence test (OLSA) or the HSM sentence test allow for the determination of speech understanding under difficult hearing conditions.

In the context of children, speech development is documented. In order to take into account that the test results depend on the age and to perform comparative evaluations, the scale of the CAPs (categories of auditory performance) was developed [55]. These CAPs describe the hearing performance and its use for communication. The categories range from 0 to 9 and reach from “no auditory sensation” up to “open speech understanding” and “use of the telephone”.

The total hearing situation in cases of bimodal and bilateral cochlear implantation can be assessed by free-field testing. Often the patients are either bimodally treated (cochlear implant and hearing aid) or bilaterally (2 cochlear implants) or have a hybrid system for electroacoustic hearing in one ear and a hearing aid in the contralateral ear (so-called combined mode). The various hearing situations have to be assessed separately and the percentage of the different hearing modalities (acoustic, electric, electroacoustic) regarding the total hearing situation must be evaluated.

### 7.2 Post-lingually deaf patients

**Figure 32:**
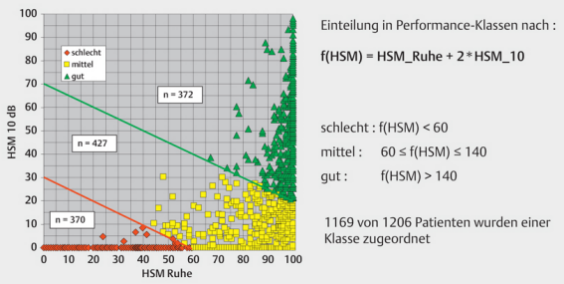
Performance categories in adult CI users

**Figure 33:**
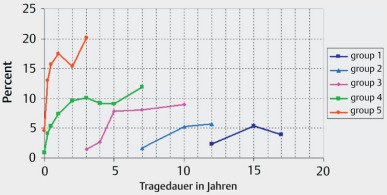
Average speech understanding in the time course depending on the implant categories, HSM sentence test S/N 10 dB (taken from: Krüger et al., 2008 [57])

**Figure 34:**
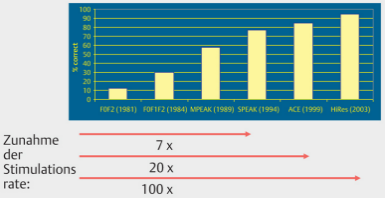
Performance improvement by increasing stimulation rates (according to A.Büchner)

In general, stable hearing results are achieved after 6–12 months. About 70% obtain an open speech understanding. However, those results are highly diverse (Figure 32 (Fig. 32)). The classification into performance categories of good, moderate, and poor is useful and oriented at the relevant prognostic parameters such as onset and duration of deafness, cognitive abilities, and cause of deafness. Also the age of life has an effect on the hearing outcome, especially when a so-called cognitive decline (see chapter 3.1) [9], [56] is observed. On average, new implant generations reveal a better speech understanding, which is mainly due to the progress in the processor technology and in particular in the stimulation rate (Figure 33 (Fig. 33), Figure 34 (Fig. 34)) [57].

### 7.3 Children

**Figure 35:**
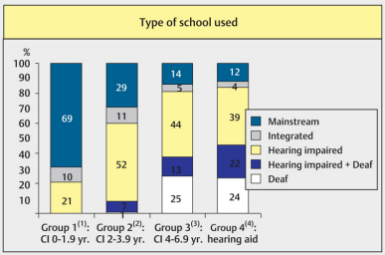
CI benefit vs. implantation age and type of school (taken from: Schulze-Gattermann et al., 2002 [58])

Speech development of children takes the time corresponding to the durations that are known for normally hearing children (in general 2–6 years). For children, a comparison with normally hearing children can be made regarding hearing and speech development. Usually, early implanted children (1st year of life) achieve very good speech development scores that nearly correspond to those of normally hearing children, especially in quiet environments. In noise, however, poorer scores are observed, which reveals that a cochlear implant does not make a child normally hearing but that it is hearing impaired. Under the aspect of the overall development, it can be summarized that, compared to normally hearing people, deficits remain even in cases of early implantation that impair the cognitive development of the brain. This is mainly due to the close interrelation between the hearing system and other brain areas and functions [31] (Figure 35 (Fig. 35)).

In cases of early implantations, about 2/3 of the children may visit regular schools [58]. The professional education is usually also significantly facilitated by cochlear implant. All professions are thus open for implanted children. Usually, however, lower school categories and professional qualifications are observed [59].

In summary, the hearing efforts of cochlear implant patients is significantly higher compared to normally hearing people. This means that a higher percentage of the cognitive capacity is used for hearing and thus the cognitive load increases. The remaining cognitive capacity for the actual learning process is hereby clearly restricted.

### 7.4 Electro-acoustic stimulation

**Figure 36:**
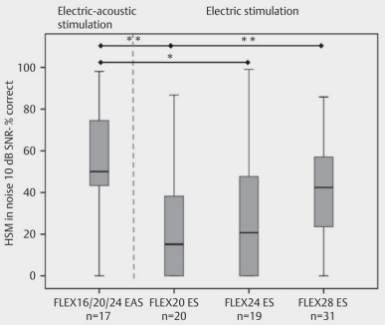
Speech understanding in noise depending on the electrode length and type of stimulation/HSM 10 dB SNR – 3 months (taken from Illg et al., Plos One 2017 [72])

Hybrid systems for electroacoustic stimulation generally lead to a clear improvement of speech understanding especially in noise, to a better directional hearing as well as a better tonal hearing (e.g. music). It is crucial to preserve residual hearing in low frequencies to use the specific advantages of acoustic hearing and to combine them with electric hearing. Limit values for the useable residual hearing in low frequencies amount to about 60 dB at 500 Hz [12], [13], [14]. The electroacoustic hearing results achieved with short electrodes are significantly better in noise compared to the hearing outcome with long electrodes and electrical stimulation alone (Figure 36 (Fig. 36)).

### 7.5 Bilateral cochlea implantation

**Figure 37:**
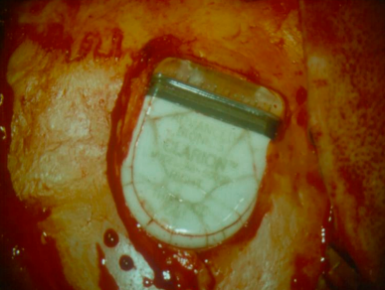
Broken implant case

The objective of bilateral cochlea implantation is an improved speech understanding in noise as well as directional hearing. Both parameters can be achieved in both ears under comparable conditions. Children may develop binaural hearing in this way (see chapter 3.4) [60] (Figure 37 (Fig. 37)).

### 7.6 Single-sided deafness

In cases of unilateral deafness, the cochlear implant may lead to a significant improvement of speech understanding in noise as well as directional hearing and tinnitus suppression. Early implantation in children may induce the development of binaural hearing [10], [11]. However, the hearing results of the implanted ear are poorer than of the normally hearing ear of the contralateral side. In cases of asymmetric hearing loss, the relative hearing gain of the implanted ear increases in the combined hearing situation. To stabilize the hearing results, repeated exercises for the implanted ear are essential.

## 8 Complications

### 8.1 Device failure (technical complications)

Device failure occurs in about 2–4% of the cases. In children they are more frequently observed than in adults which is mainly due to a higher incidence of external forces. The continuous technical improvement of the implant, in particular since the introduction of titanium cases by nearly all manufacturers, a clear reduction of the cumulative failure rate (percentage of all implant defects over a defined observation time) could be achieved [61]. Beside complete, also partial technical failures may occur, as for example the breakdown of an electrode contact. Re-implantation is indicated when the hearing performance is significantly impaired. Intermitting failures are difficult to assess technically as well as soft failure, i.e. the patient reports convincingly about hearing deterioration but a defect cannot be verified with the available technical means.

When a device failure is observed and confirmed, re-implantation should be performed as soon as possible. This is especially true for children with implant in only one ear [61], [62].

### 8.2 Medical complications

Medical complications may occur during or after surgery.

#### 8.2.1 Intraoperative complications

Intraoperative complications mainly occur as damage of the facial nerve, the sigmoid sinus, the internal carotid artery, or the ossicular chain. Further complications are injuries of the external wall of the auditory canal, the tympanic membrane, and the dura.

In general, they can be avoided by an adequate surgical technique. A low complication rate reflects a high quality standard of cochlear implantation and sufficient training due an adequate minimum number of surgeries performed per year [36], [45], [63], [64] (Table 4 (Tab. 4)).

#### 8.2.2 Postoperative complications

**Figure 38:**
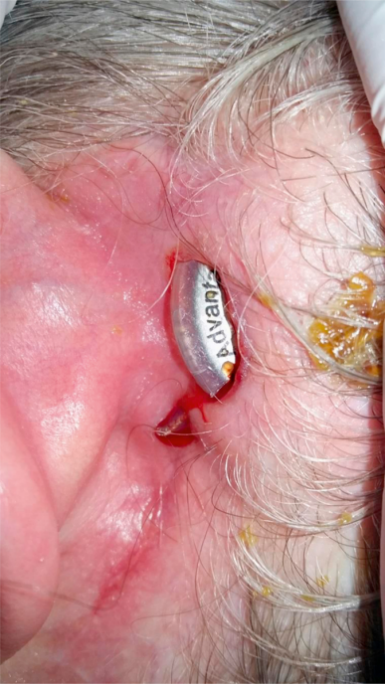
Necrotic skin over the implant

The difference is made between severe and mild complications. In general, those are either acute complications such as infections, postoperative bleedings, vertigo, or inner ear damage in the context of cochlear implantation with hearing preservation. In cases of late complications, usually long-term complications are observed. Those are among others the migration of the implant or the electrode when they are insufficiently fixed, e.g. without bone bed or without fixation of the electrode, thinning out of the skin covering the implant sometimes with perforation of the skin and infection (Figure 38 (Fig. 38)), irritation of the facial nerve in the context of advanced otosclerosis, obliteration of the cochlea in the context of labyrinthitis, or meningitis occurring after implantation [65]. An overview is given in Table 5 (Tab. 5).

Mild complications can mostly be treated conservatively. Those are also hearing deterioration with increased impedance and increased stimulus threshold, e.g. as consequence of labyrinthitis. Interestingly, those changes may also be observed in the context of overstimulation of the hearing nerve, e.g. with a very short pulse width and high rate of stimuli sequences. Hereby, the interruption of stimulation, the administration of corticosteroids as well as a careful reactivation of the stimulation are usually suitable measures to restore the stimulation capacity.

Severe complications require surgical revision, for example for re-fixation of the implant and the electrode, dislocation of the implant in cases of skin defect and according plastic measures in the sense of e.g. local rotation of the temporal muscle. For meningitis prophylaxis, vaccination against *Pneumococci* and *Haemophilus influenzae* is recommended since CI users have an increased risk. In children, specific risks must be considered that may have severe consequences (Table 6 (Tab. 6)).

**Figure 39:**
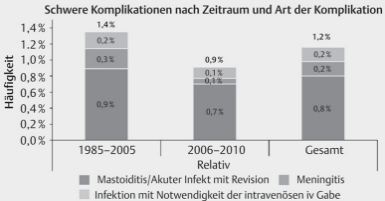
Severe complications after cochlear implantation. Significant decrease of the incidence after modification of the surgical technique (taken from: Stolle et al., 2014 [36]).

Complications require an adequate management that must be controlled by the cochlear implant surgeon. Continuous improvement of the surgical technique led to a relevant reduction of the complication rates (Figure 39 (Fig. 39)).

**Figure 40:**
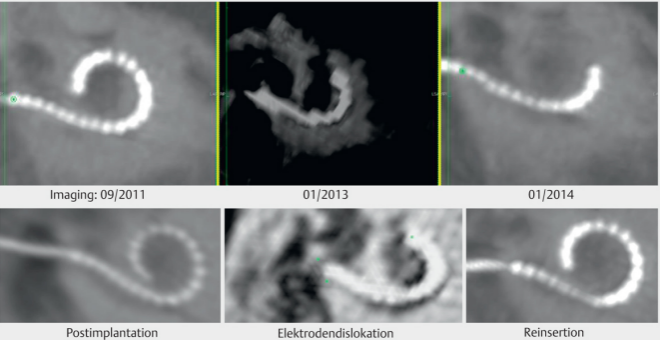
Electrode migration and re-insertion

With 6.9%, the percentage of inflammatory complications is clearly higher than in adults as well as the rate of electrode migration, which occurs in particular in atraumatic lateral wall electrodes (Figure 40 (Fig. 40)). It becomes obvious by hearing loss as well as missing NRT responses to the electrode contacts that have left the cochlea. In general, surgical revision with re-insertion of the electrode and adequate fixation is required (see chapter 4.1 and Figure 20 (Fig. 20)).

### 8.3 Re-implantation

When re-implantation is performed for technical update with replacement by a modern implant, this surgery usually leads to a better hearing result especially in co-called bad performers (see chapter 4.4.4 and Figure 30 (Fig. 30) and Figure 33 (Fig. 33)).

## 9 Research and future development

### 9.1 General aspects: bionic hearing

**Figure 41:**
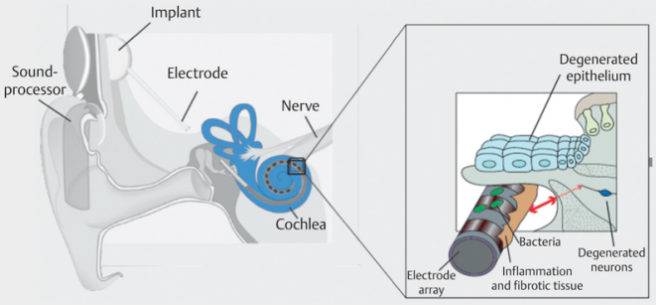
Electrode-nerve interface today

Rapid advances of cochlear implant development led to the good results of hearing rehabilitation that are achieved nowadays. However, it must be stated critically that not all patients reach open speech understanding, especially in noise, and not all children achieve a near to normal hearing and speech development. This is due to several factors. Beside the already mentioned cognitive and biographic parameters, those are mainly concomitant disease and additional disabilities. Most important, however, is the state of the hearing nerve and thus the electrode-nerve interface (Figure 41 (Fig. 41)).

The relatively wide distance from the stimulus electrode to the hearing nerve leads to an important electric field spread and consecutively poor electric channel separation. This means that current electrode systems can only realize 6–8 separated channels.

The objective of future development is the realization of the bionic ear with a substantial restoration of hearing by simulating physiological hearing with technical solutions.

Relevant elements of this bionic ear are an improved electrode-nerve interface for restoration of a near to normal physiological stimulation pattern of the hearing nerve, the regeneration of the peripheral hearing system by biological therapies, and the optimal use of the created information transmission channels by an adequate speech processing strategy.

### 9.2 Electrode-nerve interface

In current cochlear implant systems, the electrode-nerve interface is mainly determined by the low number of realized electrode contacts on the electrode carrier on the one hand, and the number of spiral ganglia cells as well as the status of the peripheral dendrites on the other hand. In addition, there are anatomical factors such as the position of the electrode in relation to the modiolus, the residual hearing and hair cells, and the ability of the patient to differentiate discrete characteristics of the provided sound signal.

An important improvement of the electrode-nerve interface that currently has a transmission capacity of about 1/10 of a compact disc player (60 vs. 700 kbit/s; Figure 2 (Fig. 2)), can be achieved by the following steps:

#### 9.2.1 Positioning near the modiolus

**Figure 42:**
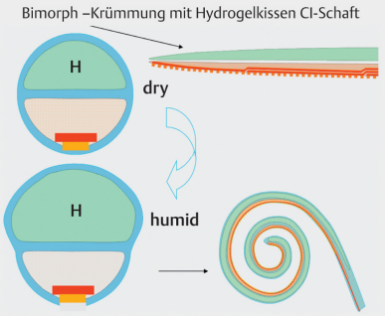
Hydrogel-based self-bending electrode (according to Doll and Stieghorst 2015)

For this purpose, pre-shaped electrode carriers are suitable that are located around the modiolus after insertion. Because of the high anatomical variability, this objective is not achieved in all cases. In this context, actively bending electrode systems are generally more appropriate to achieve this aim. After insertion, the electrodes modify their form, e.g. by temperature increase of a nitinol wire and triggering the memory effect of polymer components that enlarge by absorbing liquid of the perilymph and thus modify the electrode in a targeted way (Figure 42 (Fig. 42)) [66].

Micro-technological procedures are able to place many more discrete electrode contacts on an electrode carrier that exploits the anatomical properties of the inner ear. By omitting additional afferent wires, an unfavorable change of the mechanical insertion properties can be avoided.

#### 9.2.2 Functionalized stimulus electrodes

**Figure 43:**
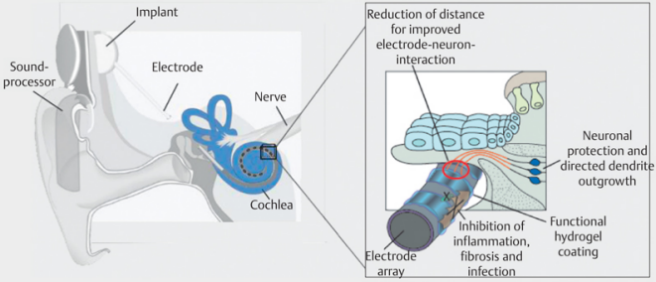
Electrode-nerve interface in the future

Even in cases of ideally positioned electrodes, still a distance between the electrode contact and the spiral ganglia cells remains in the spiral canal as well as the modiolus. Bridging would only be possible by regeneration of the peripheral hearing nerve fibers, the so-called dendrites. The application of nerve growth factors that are ideally released by the surface of the electrode and thus achieve a concentration gradient could lead to a growth of the dendrites in direction of the electrode surface (Figure 43 (Fig. 43)). This could already be confirmed in animal experiments. According surface structures of the electrode contacts may create optimal preconditions for docking nerve cells to the electrode surface in the nanoscale range. Electrode stimulation allows maintaining and using this effect [67].

#### 9.2.3 Bio-hybrid electrodes

The auto-production of nerve growth factors within the cochlea is an important factor to maintain the therapeutic effect. This might be achieved by stem cell transplantation in the inner ear. The stem cells can differentiate in the biological milieu of the inner ear and take up the auto-production of the nerve growth factors.

**Figure 44:**
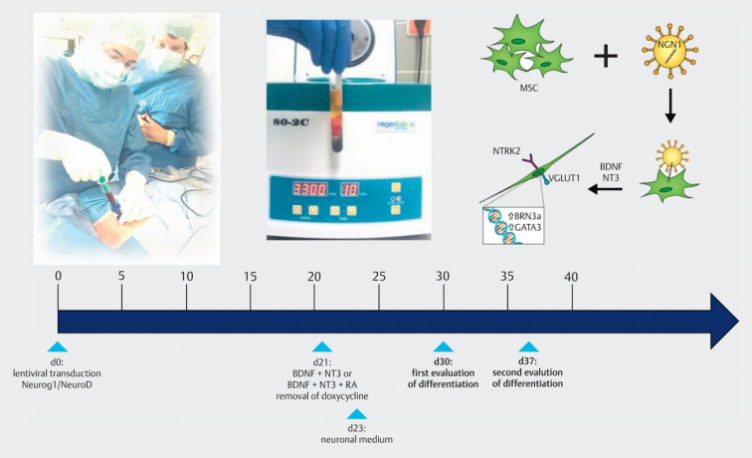
Biohybrid electrodes for stem cell transplantation into the cochlea

Stem cell transplantation can be performed by so-called bio-hybrid electrodes. After gaining the stem cells from the bone marrow of the sternum, they are applied together with the bio-hybrid electrodes onto the electrode surface by means of polymer and the electrode is then inserted carefully into the scala tympani (Figure 44 (Fig. 44)) [68].

The secretion of growth factors is also important for preservation of residual hearing. Even gene transfer by introducing nanoparticles could be imagined. Those nanoparticles would transport the correct DNA and support the remaining hair cells in their function.

#### 9.2.4 Intraneural electrodes – auditory nerve implant

**Figure 45:**
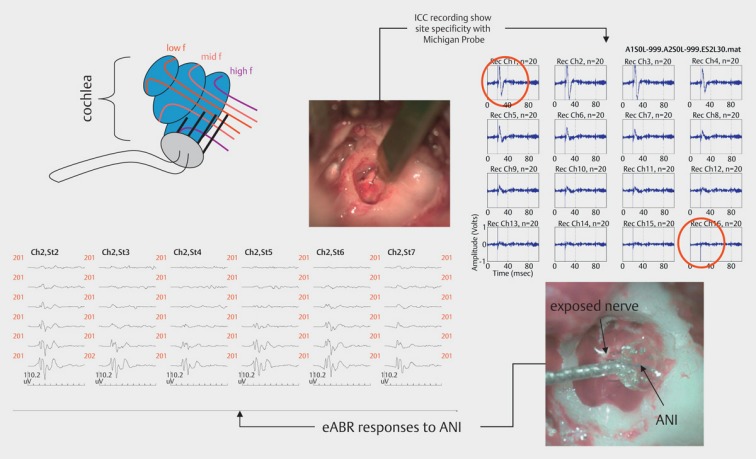
Auditory Nerve Implant (ANI). Direct stimulation of the hearing nerve triggers tonotopic stimulation in the inferior colliculus.

Alternatively, intraneural electrode systems can be developed. The electrode contacts are directly positioned in the hearing nerve and an improved channel separation is achieved. According electrode systems with high contact density are currently developed (Figure 45 (Fig. 45)).

### 9.3 Robotic systems

Robotic systems are used in minimally invasive cochlear implant surgery and contribute to an improved insertion and positioning of the electrode systems in the cochlea. Because of the evident high inter-individual variability of anatomical cochlear parameters such as the length of the external wall (variation between 35 and 46 mm), these factors may be addressed by a precise insertion of the electrode. Beside the selection of suitable trajectories, the optimal insertion of the electrode as well as the 3-dimensional control of the other parameters play a crucial role [69] (Figure 24 (Fig. 24)).

### 9.4 Speech coding strategies

The improved electrode-nerve interface allows new and better possibilities of speech processing strategies. Those are algorithms that translate the acoustic signal into a logical sequence of electrical pulses for the cochlear implant system. In terms of a significant improvement of the electrode-nerve interface with a higher number of electrically separated channels, other speech coding strategies can be applied that aim at an increase of the transmitted information, a spectral contrasting, and a simulation of physiological stimulation patterns of the hearing nerve. Suitable modelling of the individual electric distribution in the cochlea allows for the best combinations of electrode contacts for stimulation.

### 9.5 Brain-computer interface and closed-loop systems

The use of objective parameters, especially of acoustically evoked compound action potentials of the hearing nerve as well as the centrally located auditory evoked potentials, currently so-called closed-loop systems are being developed, in which additional registration electrodes are placed for example above the auditory cortex. The implant serves at the same time as sensor (theranostic implant).

The obtained EEG signals can be used for fine tuning of speech processing with selection of appropriate parameters and for the support in difficult acoustic situations. This is of high importance especially for children. Because of the higher complexity of speech coding algorithms, it becomes more and more important also in adults and it will substitute the currently manual programming.

### 9.6 Multimodal stimulation of the inner ear

The increasing number of patients that are treated with residual hearing makes it necessary to adequately use the acoustic residual hearing in combination with the electrical hearing. For this purpose, electrode systems with integrated mechanical actuators or an optoacoustic fiber actuator are developed. They can be adjusted to the individual functional state of the inner ear and allow an optimized usage of the cochlear reserve.

In order to achieve a synchronized stimulation of the cochlea for the acoustic and electrical stimulus, it is necessary to timely harmonize both stimuli. Additionally, the mechanical stimulus should be coupled ideally into the cochlea to avoid coupling problems. Hereby, the use of multimodal stimulation systems is suitable; here, a mechanical stimulator may be also an integral part of the electrode system. Further, actuators on electromagnetic or piezoelectric basis are possible. Those so-called electromechanical stimulations open new possibilities for the use of the cochlear reserve and may adjust to a changing residual hearing so that ideally re-implantation of the patient can be avoided.

#### 9.6.1 Optoacoustic stimulation

Stimulation with high-energy optical short pulses via laser systems leads to a stimulation of the hair cells in the inner ear. The basis is the so-called optoacoustic effect based on thermo-elastic expansion. The applied laser pulse leads to a short-term temperature increase of the biological tissue and thus the development of a mechanical pulse. This is the adequate stimulus for the hair cells to be activated.

This optoacoustic effect might also be used for electromechanical stimulation that was described above [70].

#### 9.6.2 Optogenetic stimulation

In contrast to optoacoustic stimulation, that does not directly activate auditory neurons, the optogenetic stimulation uses the sensitization of the neuronal tissue by initially applied pigments. They can permanently be generated via genetic manipulation so that the treated spiral ganglia cells are sensitized for optical stimuli [71].

### 9.7 Invisible hearing – fully implantable cochlea implant systems

Advances in battery and microphone technology allowed fully implantable hearing systems. The energy supply is performed by transcutaneously charged batteries. The sound reception occurs transcutaneously. If needed, an external speech processor can be coupled. Currently battery lifetimes of about 10–15 years are considered as being realistic. The patient gains further freedom of action and loses the stigma of disability. One disadvantage is the restriction to software updates to participate in the technological progress outside the re-implantation period.

### 9.8 Cochlear implants as personal communicator

By including the cochlear implant in a superordinate communication system, the possibilities of audiotechnology and telecommunication can be fully used for the cochlear implant. The control is performed for example via Bluetooth.

## 10 Conclusion

Rapid technological progress of cochlear implants makes the development of the bionic ear more and more realistic. Improved hearing results will lead to the fact that more patients benefit from this technology. Currently there are about 1 million candidates for cochlear implantation only in Germany, actually about 50,000 are implanted. To reach all patients, according advances in the fields of technology and biology are necessary. Those technical developments will allow performing surgery in a minimally invasive way, if possible under local anesthesia, during a short-term intervention. Suitable automated procedures will allow for an adequate fitting and thus create the preconditions for hearing that is as natural as possible.

## Notes

### Competing interests

The author declares that he has no competing interests.

## Figures and Tables

**Table 1:**
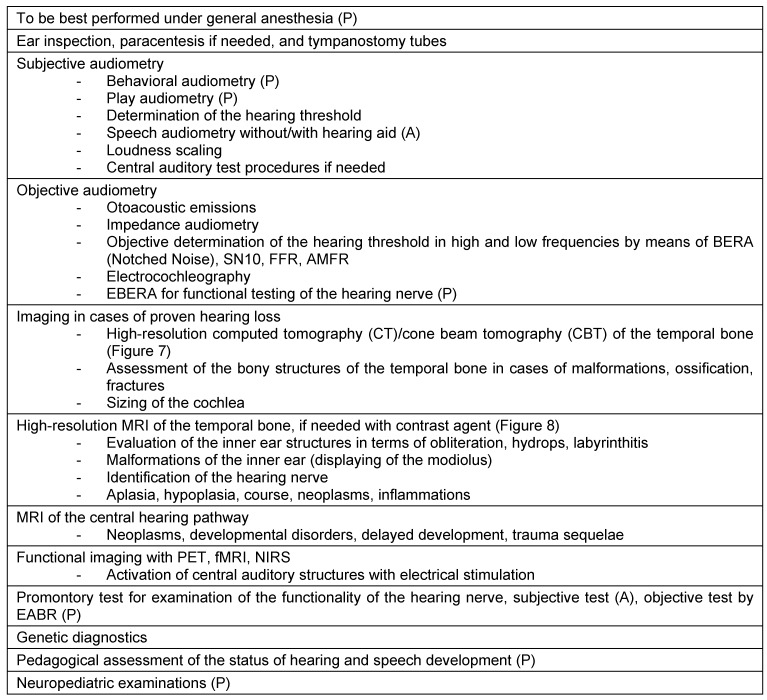
Preoperative diagnostics for indication of cochlear implantation in adult (A) and pediatric (P) patients

**Table 2:**
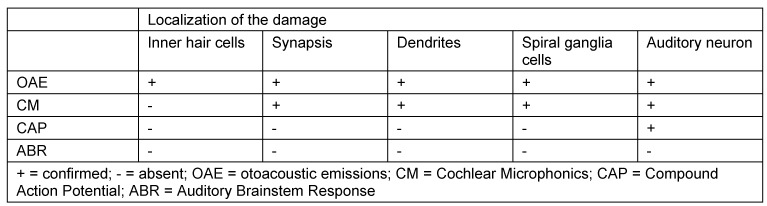
Constellation of findings in cases of perisynaptic audiopathy

**Table 3:**
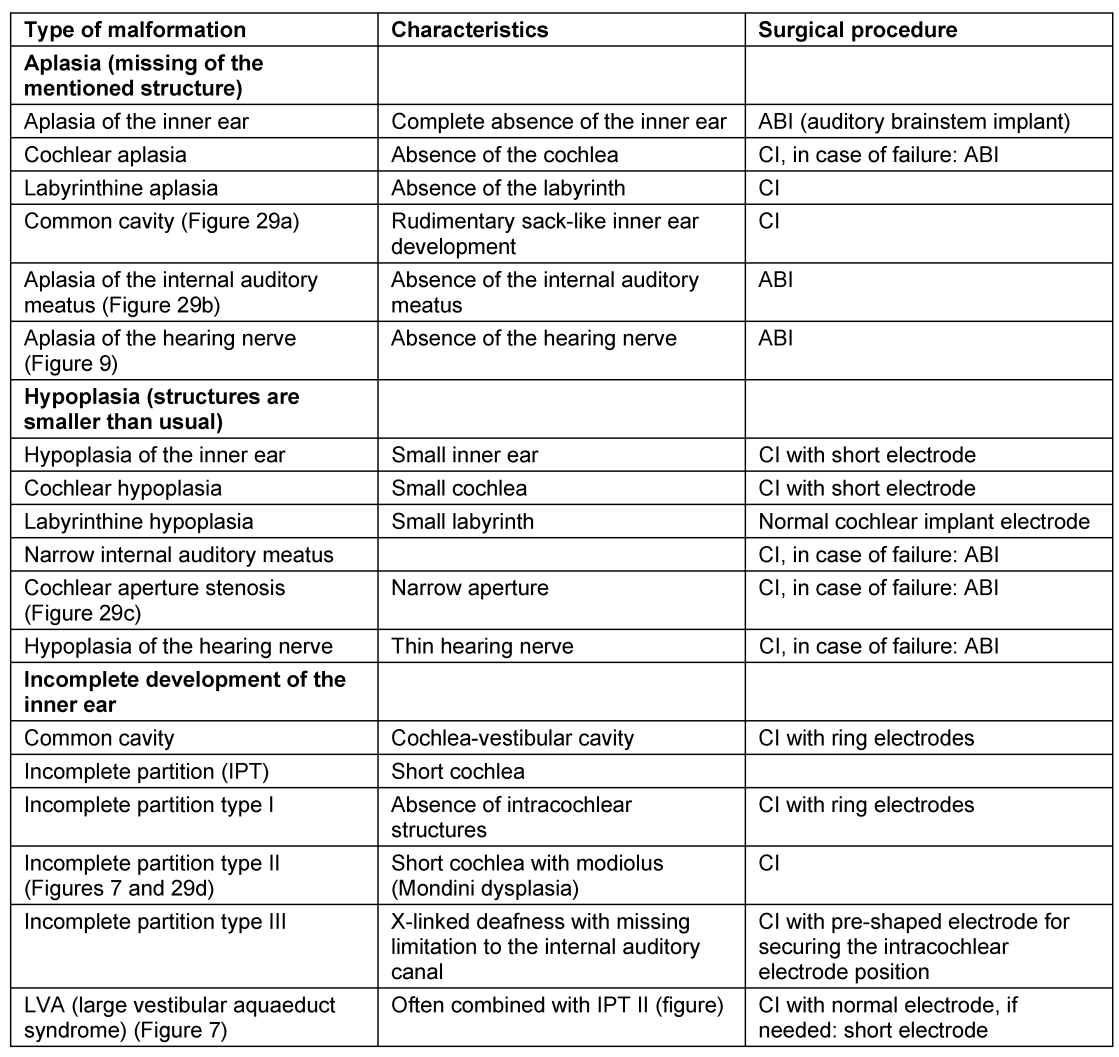
Malformations of the inner ear and the temporal bone and cochlear implantation based on Sennaroglu and Saatci, 2003 [42]

**Table 4:**
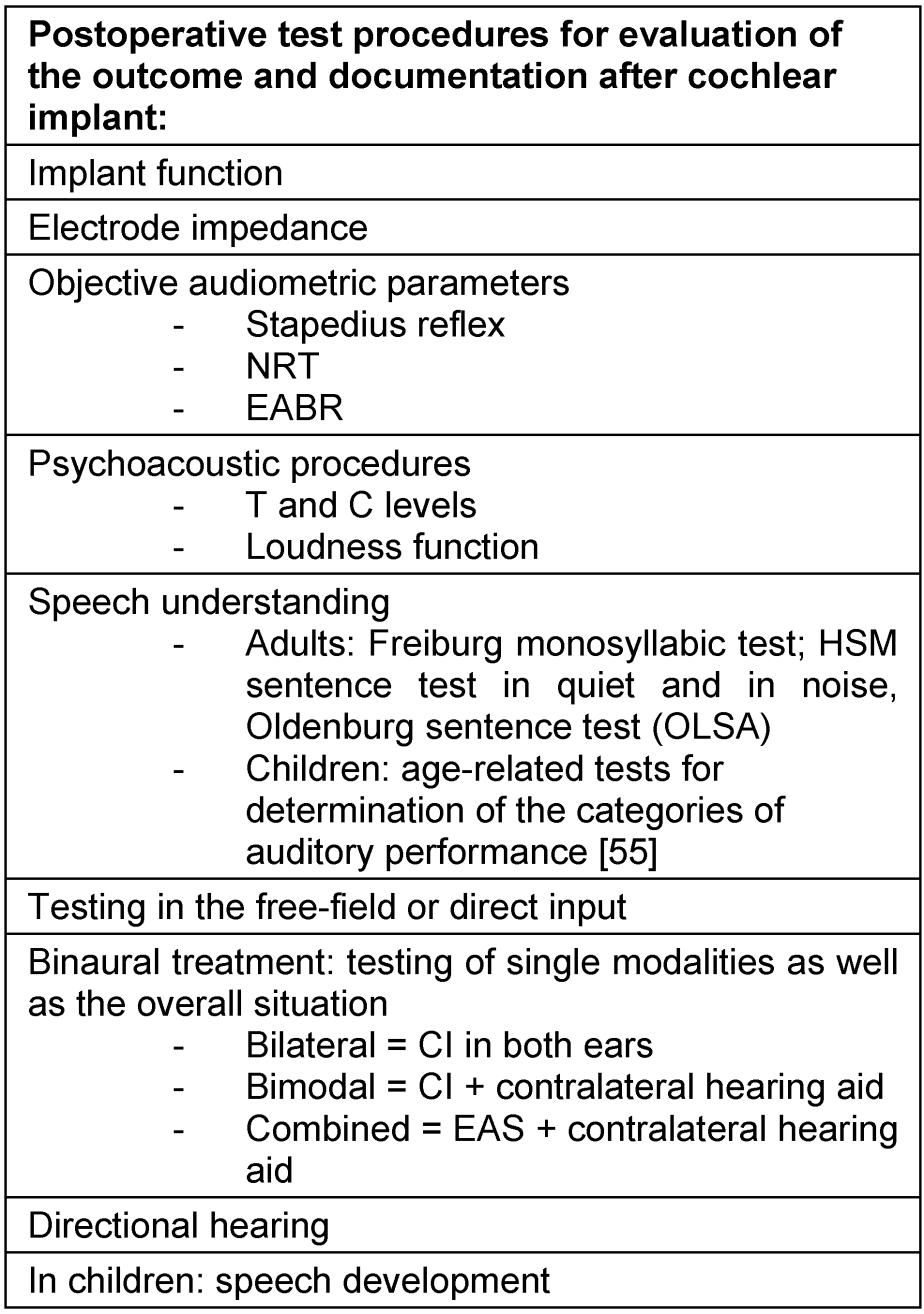
Test procedures for assessment of hearing performance

**Table 5:**
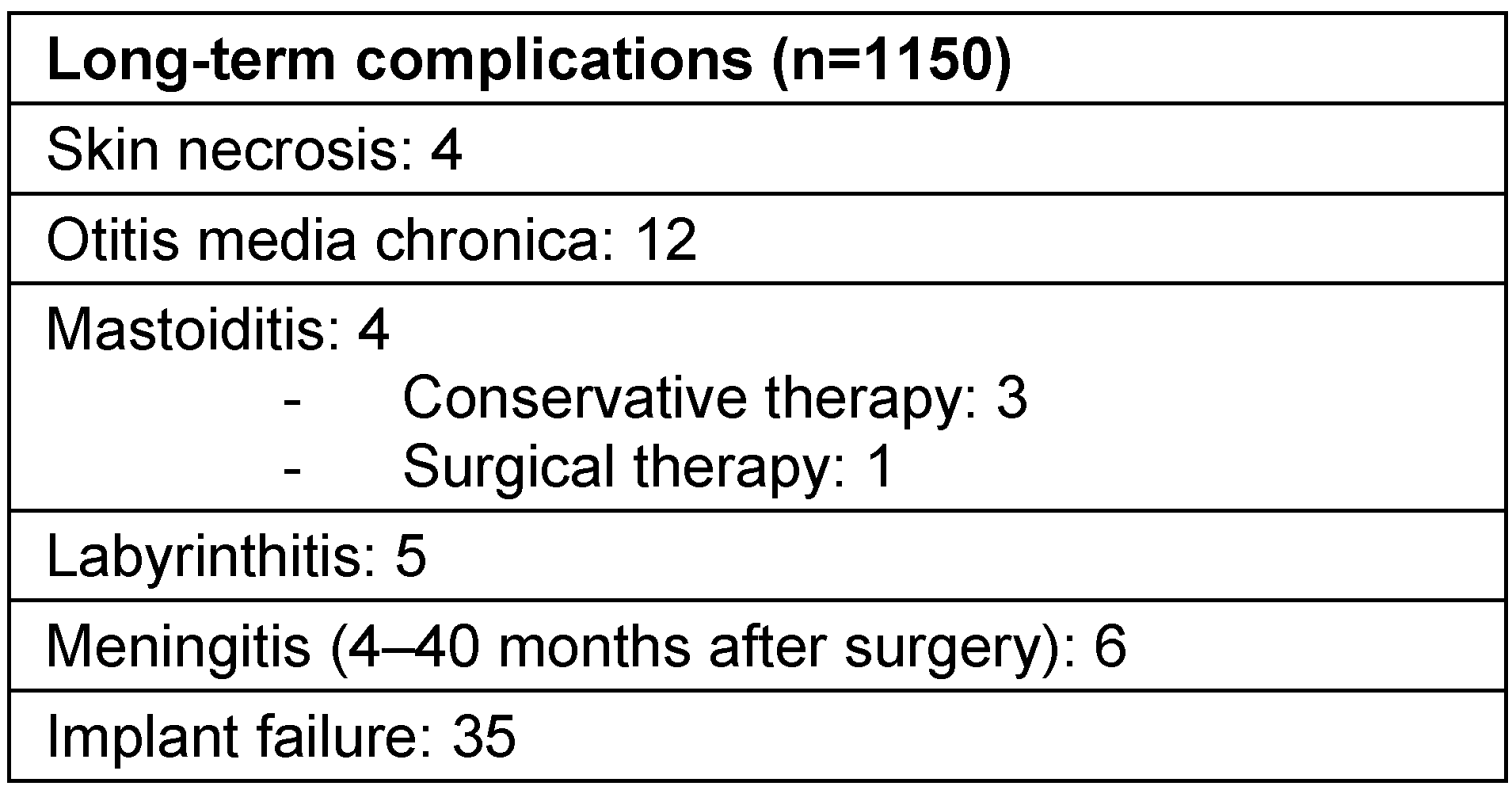
Postoperative complications

**Table 6:**
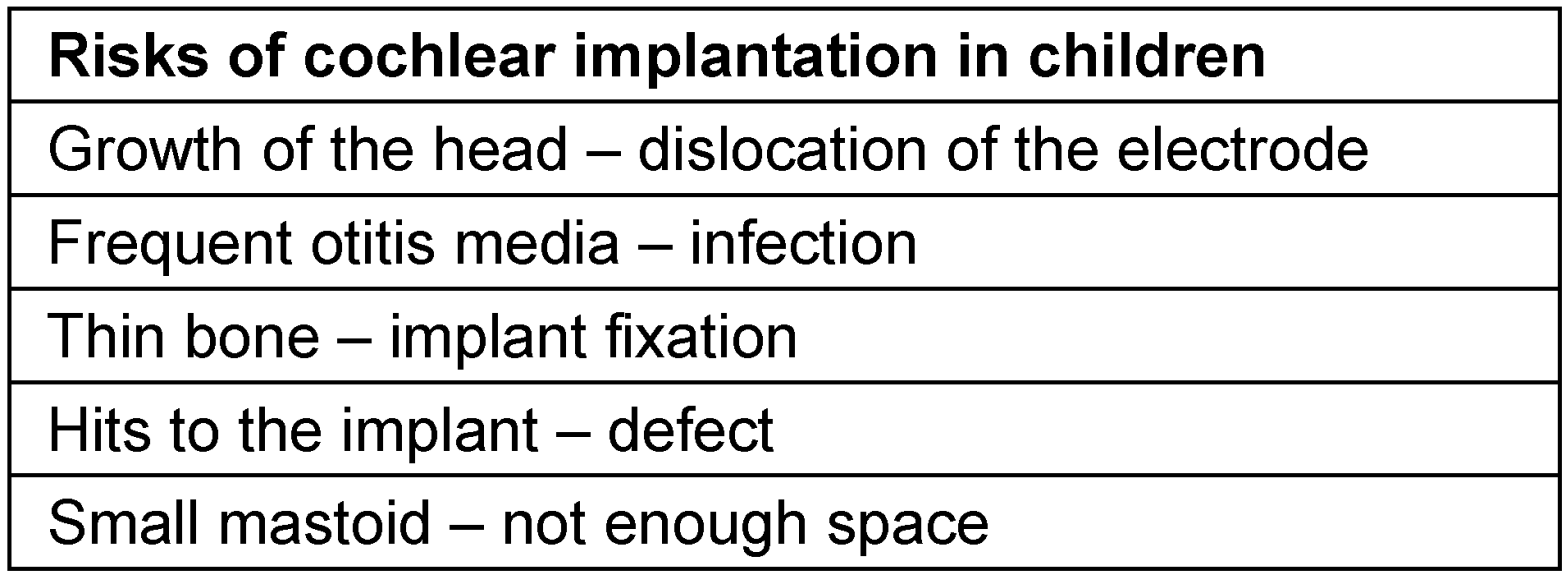
Risks in children
